# *Culex pipiens* and *Culex restuans* mosquitoes harbor distinct microbiota dominated by few bacterial taxa

**DOI:** 10.1186/s13071-016-1299-6

**Published:** 2016-01-13

**Authors:** Ephantus J. Muturi, Chang-Hyun Kim, Jeffrey Bara, Elizabeth M. Bach, Madhura H. Siddappaji

**Affiliations:** Illinois Natural History Survey, University of Illinois, 1816 S. Oak St., Champaign, IL 61820 USA; Department of Biology, University of Louisville, Louisville, KY 40292 USA

**Keywords:** *Culex pipiens*, *Culex restuans*, Microbiota

## Abstract

**Background:**

Mosquitoes host diverse microbial communities that influence many aspects of their biology including reproduction, digestion, and ability to transmit pathogens. Unraveling the composition, structure, and function of these microbiota can provide new opportunities for exploiting microbial function for mosquito-borne disease control.

**Methods:**

MiSeq® sequencing of 16S rRNA gene amplicons was used to characterize the microbiota of adult females of *Culex pipiens* L. and *Cx. restuans* Theobald collected from nine study sites in central Illinois.

**Results:**

Out of 195 bacterial OTUs that were identified, 86 were shared between the two mosquito species while 16 and 93 OTUs were unique to *Cx. pipiens* and *Cx. restuans*, respectively. The composition and structure of microbial communities differed significantly between the two mosquito species with *Cx. restuans* hosting a more diverse bacterial community compared to *Cx. pipiens. Wolbachia* (OTU836919) was the dominant bacterial species in *Cx. pipiens* accounting for 91 % of total microbiota while *Sphingomonas* (OTU817982) was the dominant bacterial species in *Cx. restuans* accounting for 31 % of total microbiota. Only 3 and 6 OTUs occurred in over 60 % of individuals in *Cx. pipiens* and *Cx. restuans*, respectively. There was little effect of study site on bacterial community structure of either mosquito species.

**Conclusion:**

These results suggest that the two mosquito species support distinct microbial communities that are sparsely distributed between individuals. These findings will allow investigations of the role of identified microbiota on the spatial and temporal heterogeneity in WNV transmission and their potential application in disease control.

**Electronic supplementary material:**

The online version of this article (doi:10.1186/s13071-016-1299-6) contains supplementary material, which is available to authorized users.

## Background

The urgent need to develop novel tools for controlling mosquito-borne diseases such as malaria, dengue, Chikungunya, and West Nile Virus (WNV) has stimulated research interest in understanding the structure and function of mosquito microbiota. These studies have ranged from field surveys on the composition and diversity of mosquito microbiota [1–5] to investigations of their impact on host fitness and response to parasitic and viral infections [6–9]. Results from these studies have revealed that adult mosquitoes host a community of natural microbiota [1–3] that contributes to host survival, reproduction, nutrition, and immunity [7, 8, 10–13]. Some endogenous microbes have also been shown to reduce vector lifespan [14, 15] and susceptibility to parasitic and viral agents [7, 16–18]. In addition, certain mosquito microbial symbionts can be genetically modified to express molecules that inhibit pathogen transmission [19–21]. These findings have provided the impetus for controlling mosquito-borne diseases through manipulation of mosquito-associated microbiota [22]. However, our limited understanding of the composition, diversity, and function of mosquito microbiota continues to be a critical barrier to practical application of microbes for the control of mosquito-borne diseases. Specifically, while the natural microbiota of malaria vectors (*Anopheles* spp.) and dengue and Chikungunya (*Aedes spp.)* vectors [1, 3, 6, 12, 23, 24] are well characterized, little is known about the natural microbiota of mosquitoes from other genera such as *Culex* and *Mansonia* [2, 4, 25, 26], despite their role as vectors of human and animal diseases such as WNV and lymphatic filariasis.

In this study we employ high throughput MiSeq® sequencing of 16S rRNA to investigate the diversity and structure of bacterial communities of adult females of *Culex pipiens* and *Culex restuans* mosquitoes collected in central Illinois from three of the dominant land-use categories across the Midwestern USA: urban woodlots, rural woodlots, and agricultural land [27]. Despite the role of the two mosquito species as the primary vectors of WNV in northern United States, little is known about the composition and structure of their microbiota. Understanding the microbiota of these two vector species will allow investigations of their influence on WNV transmission and provide new insights into how these microbiota may be exploited for disease control.

## Methods

### Study sites

**Fig. 1:**
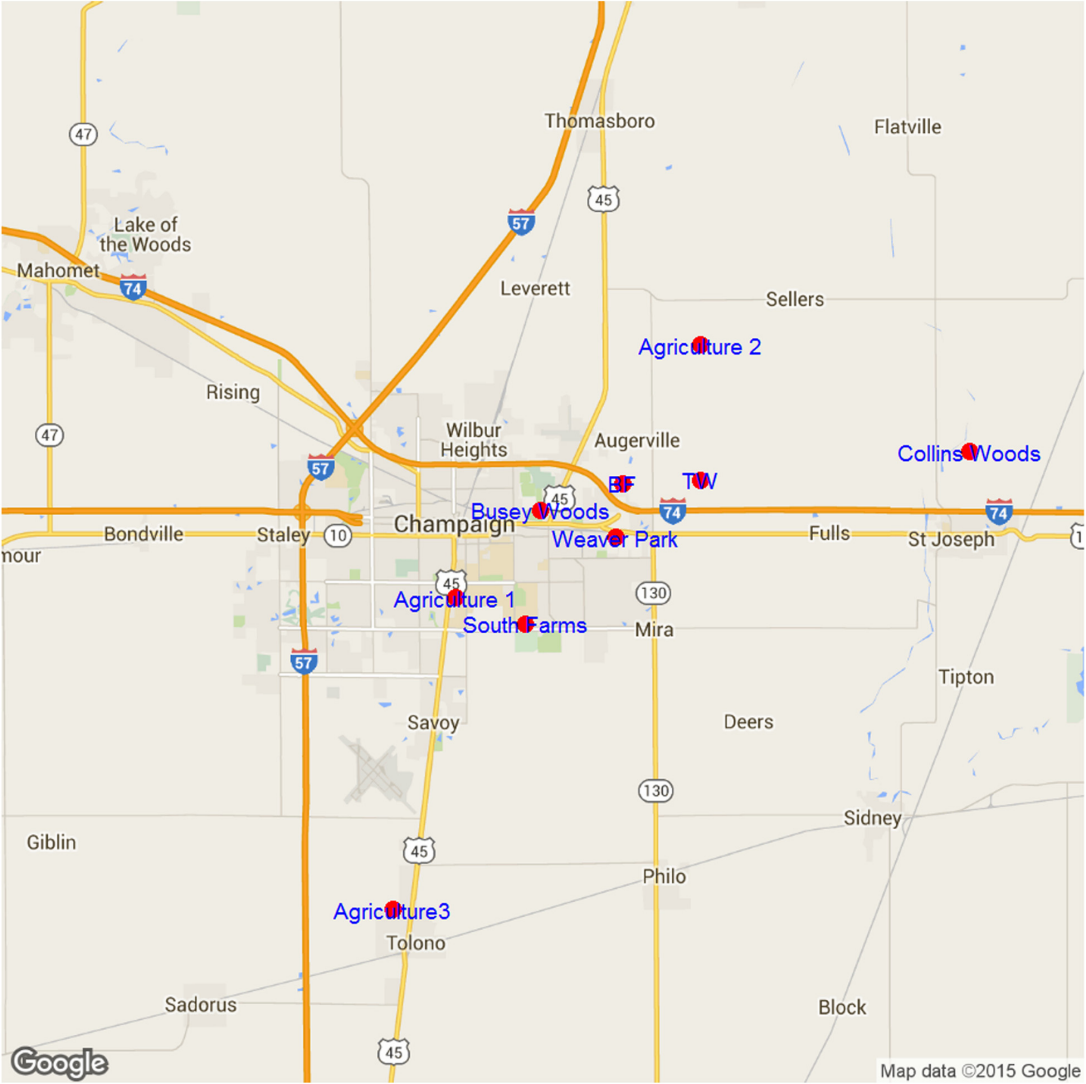
Map of Champaign County showing the location of the nine study sites. TW and BF represent the Trelease Woods and Brownfield Woods study sites respectively

The studies were conducted in Champaign County, Illinois where WNV is well established [27]. Nine study sites representing three urban woodlots (Weaver Park, Busey Woods, South Farms), three rural woodlots (Collins Woods, Trelease Woods, Brownfield) and three agricultural sites (sites 1-3) were selected for this study (Fig. 1). These sites were chosen to reflect the dominant land-use categories common to the Midwestern United States. GPS coordinates for these sites are presented in Additional file 1: Table S1. Agricultural sites are row crop farms rotated with corn and soybeans. At the time of sampling, these sites were occupied by rows of corn plantations. Weaver Park is a 24.28-ha land located approximately 4.35 km NE of University of Illinois at Urbana-Champaign (UIUC) campus. The park contains an in-progress 2.02-ha woodland/savanna restoration, 14.16-ha planted with prairie and native grasses and a watershed management wetland. Busey Woods is a 23.88-ha bottomland Oak-Hickory forest located at the north end of Crystal Lake Park approximately 1.6 km NE of UIUC campus. South Farms is a 8.15-ha forest located approximately 2.41 km SE of UIUC campus. The site is characterized by high canopy trees composed of maple, sycamore, pine, oak, and a dense understory of grasses and invasive Amur honeysuckle (*Lonicera maackii*).

Collins Woods is a 5.3-ha second growth deciduous forest with a livestock grazing history. The site is located about 19.31 km NE of the UIUC campus. The eastern half is a mix of older “wolf” trees and woody successional species. The western half is a more mature oak/hickory/ash/osage orange woods with a small area of old river oxbow bottom land. Bush honeysuckle (*Lonicera spp*.) dominates the understory in many areas.

Trelease Woods is a 28.8-ha old growth deciduous forest located approximately 9.66 km northeast of the UIUC campus. The site is characterized by a high closed canopy with a moderately dense understory and primarily composed of mature oak, ash, hackberry, and maple species. A detailed description of this study site is provided elsewhere [28].

Brownfield Woods is a 26.1-ha “virgin” deciduous upland forest located in Urbana, Illinois, approximately 6.44 km NE of the UIUC campus. It is primarily a mature oak/ash/maple forest with a high, closed canopy and fairly open understory. Sugar maple has rapidly become the dominant tree species. The woods are a remnant of a much larger prairie grove that was present at settlement times. A small creek that is fed by runoff and field tiles, runs diagonally through the woods. Agricultural land borders the west side and across the road on part of the east side.

### Mosquito collection

Adult females of *Cx. pipiens* and *Cx. restuans* were collected from the nine study sites once every two weeks between June 19, 2014 and September 10, 2014. The collections were made using Centers for Disease Control (CDC) gravid traps (John W. Hock Co., Gainesville, FL) baited with 5-day old grass infusion. Attempts were made to distinguish *Cx. pipiens* and *Cx. restuans* mosquitoes based on the presence of two pale spots that are present on the scutum of *Cx. restuans* but are absent in *Cx. pipiens* [29]*.* However, this effort was abandoned because most samples had lost their scales and therefore mosquito samples identified as *Cx. pipiens/Cx. restuans* were classified as *Culex* spp. (see Additional file 1: Table S1) and stored at -80 °C until further processing.

### DNA extraction and 16 s rRNA library preparation

One hundred and forty seven (147) randomly selected non-blood fed mosquito samples representing all the nine study sites were retrieved from -80 °C freezer, rinsed three times in sterile water and surface disinfected in 70 % ethanol for 10 min. The ethanol was then removed by rinsing the mosquitoes five times in sterile water and once in dilute (0.8 %) saline solution [3]. DNA from mosquito samples was extracted using MoBio PowerSoil isolation kit (MO BIO, Carlsbad, CA). Briefly, individual mosquitoes were ground in 500 μL of solution from PowerBead tubes of MoBio PowerSoil kit and the entire homogenate was used for DNA extraction as per manufacturer’s recommendation. DNA was quantified using NanoDrop 1000 (Thermo Scientific, Pittsburg, PA) and its quality assessed using Agilent 2100 Bioanalyzer (Agilent Technologies, Santa Clara, CA). A portion of DNA samples was used for mosquito species identification using real-time polymerase chain reaction [30]. These yielded 54 and 93 *Cx. pipiens* and *Cx. restuans* samples respectively. For bacterial characterization, we targeted the V3-V5 hypervariable region of the 16S rRNA gene (694 base pairs) using the following primer set: forward 5`-CCTACGGGAGGCAGCAG-3` and reverse 5`-CCGTCAATTCMTTTRAGT-3` [31].

Library preparation and sequencing were conducted at the W. M. Keck Center for Comparative and Functional Genomics at the University of Illinois at Urbana-Champaign (Additional file 2: Appendix S1). Briefly, DNA of each mosquito sample was amplified using the above primer set on the Fluidigm® microfluidics quantitative PCR platform with appropriate linkers and sample barcodes. The final Fluidigm® libraries were pooled in equimolar ratio for sequencing. The final denatured library pool was spiked with 10 % non-indexed PhiX control library (Illumina®) and sequenced by 2 x 300 nt paired-end sequencing on the Illumina® MiSeq® V3 Bulk system. The PhiX control library provides a balanced genome for calculation of matrix, phasing and pre-phasing, which are essential for accurate base-calling. The libraries were sequenced from both ends of the molecules to a total read length of 300 nt from each end. Cluster density was 964 k/mm^2^ with 85.9 % of clusters passing filter.

We present the results for the forward reads only because reverse reads, which are not independent from the forward reads, showed the same patterns among samples (see Additional file 3: Figure S1, Additional file 4: Figure S2 and Additional file 5: Table S2). This is expected because single-end reads are known to be sufficient to observe the same relationships among samples that are revealed with paired-end reads [32].

### Illumina OTU analysis and statistics

The QIIME version 1.9.0 pipeline [33] was used to demultiplex and quality filter the forward and reverse reads using defaults [34]. Barcodes and primer sequences were removed using Fastx toolkit operated in QIIME. The operational taxonomic units (OTUs) were picked and identified at 97 % similarity using Greengenes reference [35] sequence database (http://greengenes.lbl.gov/cgi-bin/nph-index.cgi) downloaded on May 2015. Due to variations in the number of sequences between samples (range 5-210,445 sequences), read depth was rarefied to 2000 reads per sample to retain adequate samples for statistical analysis and to standardize the sampling effort [36]. OTUs accounting for <0.005 % of the total sequences were discarded to reduce the problem of spurious OTUs which may result from random sequencing errors and are likely to overestimate the overall diversity [6, 34]. Alpha diversity metrics including Shannon diversity index, observed species, chao1, and evenness were generated in QIIME and analysis of variance (ANOVA) with Tukey adjustments was used to test the effect of site and mosquito species on these indices using SPSS statistical package (IBM Inc.). A subset of the data containing equal numbers of *Cx. pipiens* and *Cx. restuans* was also analyzed, and overall results were similar to those of data containing all samples indicating that sample size disparities did not contribute to observed differences in microbial diversity and richness between the two mosquito species. Bacterial communities were visualized using non-metric multi-dimensional scaling (NMDS) plots generated using vegan package [37] in R [38]. Non-parametric multivariate community analysis including indicator species analysis and Multi-Response Permutation Procedures (MRPP) were conducted using PC-ORD version 6.08 [39]. MRPP was used to test for differences in microbial communities between mosquito species and study sites while indicator species analysis was used to identify bacterial species that are strongly associated with each of the two mosquito species. Indicator values range from 0 to 1 with a value of 1 indicating that the species occurs in all samples of a treatment and are specific to those samples [40].

## Results

### Bacterial species composition differs between *Cx. pipiens* and *Cx. restuans*

We analyzed the V3-V5 hyper-variable region of 16S rRNA gene to estimate the composition and structure of bacterial communities of field-collected adult females of *Cx. pipiens* and *Cx. restuans*. A total of 50 and 84 *Cx. pipiens* and *Cx. restuans* females, respectively had at least 2000 16S rRNA sequences and were retained for further analyses. After rarefaction and quality filtering, 268,000 16S rRNA sequences were retained and classified into Operational Taxonomic Units (OTUs). In total, there were 195 bacterial OTUs belonging to 9 phyla (including one unclassified phyla) (Table 1) and 54 families. Of the 195 OTUs, 188 (96.4 %) were classified to the family level while only 135 (69.2 %) OTUs were assigned to the genera rank. Only 3 (OTU836919-*Wolbachia*, OTU817982-*Sphingomonas* and OTU6118-*Wolbachia*) and 6 OTUs (OTU836919-*Wolbachia*, OTU817982-*Sphingomonas*, OTU4323871-*Methylobacterium komagatae*, OTU573035-*Alicyclobacillus*, OTU4363508*-Sphingomonadaceae* and OTU17329- *Sphingomonadaceae*) occurred in over 60 % of individuals in *Cx. pipiens* and *Cx. restuans* respectively. Overall, OTU836919-*Wolbachia* and OTU817982-*Sphingomonas* occurred in 98 % and 90 % of all samples. The phylum *Proteobacteria* was the most dominant in both mosquito species accounting for 99 % of total microbiota in *Cx. pipiens* and 81 % of total microbiota in *Cx. restuans* (Table 1). *Alphaproteobacteria* and *Gammaproteobacteria* were the most dominant subdivisions of phylum *Proteobacteria* accounting for 94 % and 4 % of total microbiota respectively in *Cx. pipiens* and 56 % and 21 % of total microbiota respectively in *Cx. restuans* (Table 1). *Firmicutes* was the second most common phylum in both mosquito species accounting for 0.57 % of total bacteria in *Cx. pipiens* and 13 % of total bacteria in *Cx. restuans* (Table 1).

**Table 1:** Phylum-level classification of bacterial communities from *Cx. pipiens* and *Culex restuans*

		*Cx. pipiens*		*Cx. restuans*	
Phylum	Class	#OTUs	Relative abundance (%)	#OTUs	Relative abundance (%)
Acidobacteria	Solibacteres	0	0.00	2	0.02
Actinobacteria	Actinobacteria	3	0.24	9	2.90
	Thermoleophilia	1	0.01	0	0.00
Bacteroidetes	Bacteroidia	0	0.00	2	0.21
	Cytophagia	1	0.01	2	0.03
	Flavobacteriia	3	0.19	2	1.48
	Sphingobacteriia	0	0.00	2	0.61
Chloroflexi	Ellin6529	1	0.01	0	0.00
Cyanobacteria	Chloroplast	0	0.00	2	0.54
	Oscillatoriophycideae	0	0.00	1	0.20
Firmicutes	Bacilli	11	0.53	15	11.23
	Clostridia	5	0.04	13	1.56
Proteobacteria	Alphaproteobacteria	32	94.37	45	55.70
	Betaproteobacteria	16	0.17	31	3.81
	Deltaproteobacteria	1	0.30	0	0.00
	Gammaproteobacteria	28	4.14	51	21.13
Spirochaetes	Spirochaetes	0	0.00	1	0.45
WPS-2	Unclassified	0	0.00	1	0.14
	Total	102	100.00	179	100.00

**Fig. 2:**
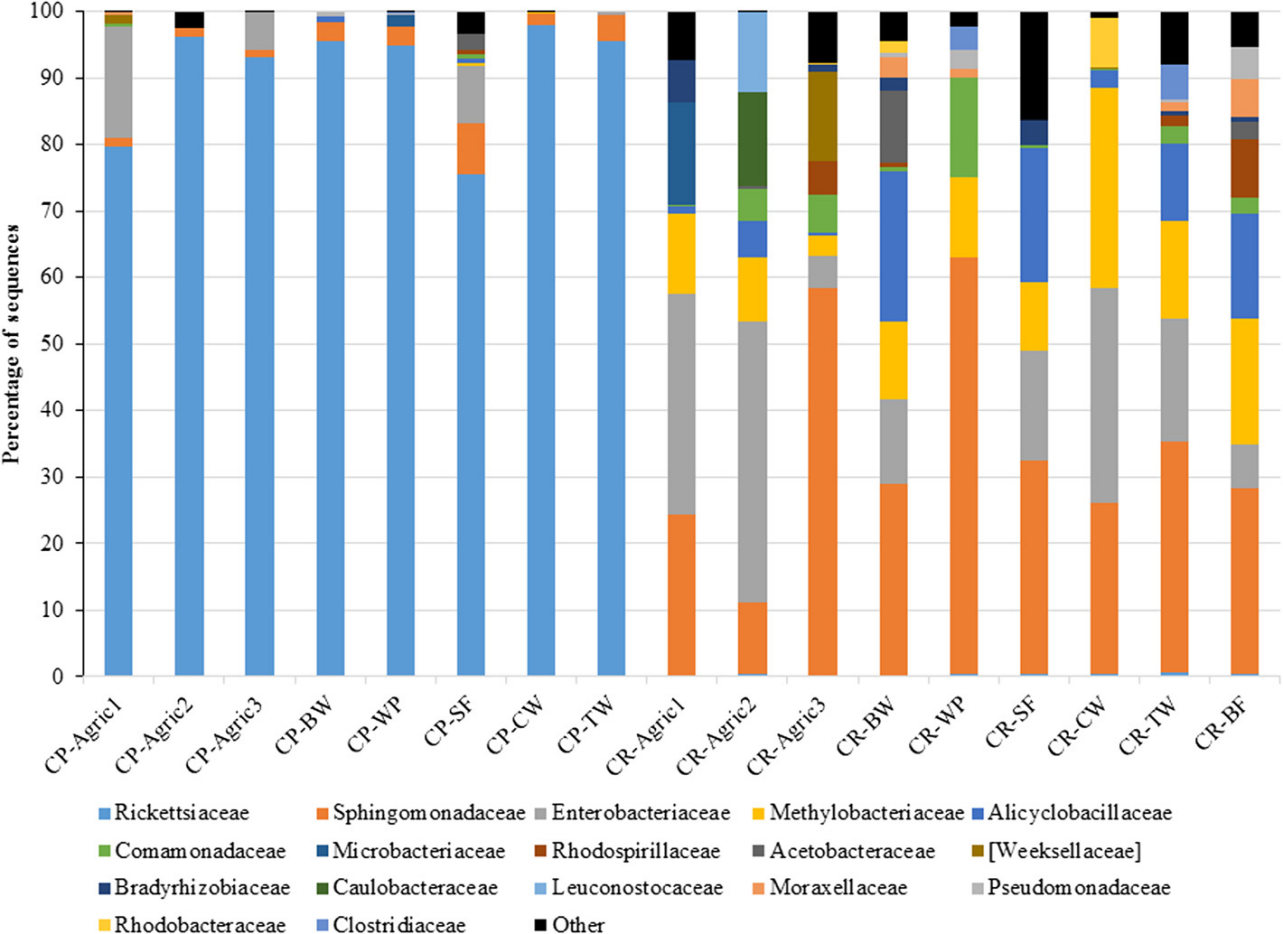
Relative abundance of the top 20 bacterial families in *Cx. pipiens* and *Cx. restuans* samples from different study sites. (CP = *Cx. pipiens*, CR = *Cx. restuans*, Agric = Agriculture, BW = Busey Wood, WP = Weaver Park, SF = South Farms, CW = Collins Woods, TW = Trelease Woods, and BF = Brownfield Woods)

At the family level, *Rickettsiaceae* accounted for 91 % of total microbiota in *Cx. pipiens* followed by *Enterobacteriaceae* (4 %) and *Sphingomonadaceae* (3 %). In *Cx. restuans*, the three most abundant families were *Sphingomonadaceae* (34 %), *Enterobacteriaceae* (19 %), and *Methylobacteriaceae* (14 %). The top 20 bacterial families across study sites represented 97–100 % of total microbiota in *Cx. pipiens* and 84–99 % of total microbiota in *Cx. restuans* (Fig. 2). The relative abundance of the 20 families varied markedly by mosquito species and study site (Fig. 2). Overall, only 9 families had a relative abundance equal to or greater than 1 % (Fig. 2).

Eighty six bacterial OTUs were shared between the two mosquito species but none were present in all samples. These shared bacterial OTUs were from four phyla: *Proteobacteria* (67 OTUs), *Firmicutes* (14 OTUs), *Bacteroidetes* (3 OTUs), and *Actinobacteria* (2 OTUs) (Additional file 6: Table S3). Sixteen bacterial OTUs were unique to *Cx. pipiens* and 93 bacterial OTUs were unique to *Cx. restuans* (Additional file 6: Table S3).

### Bacterial species diversity and richness is higher in *Cx. restuans* than in *Cx. pipiens*

**Fig. 3:**
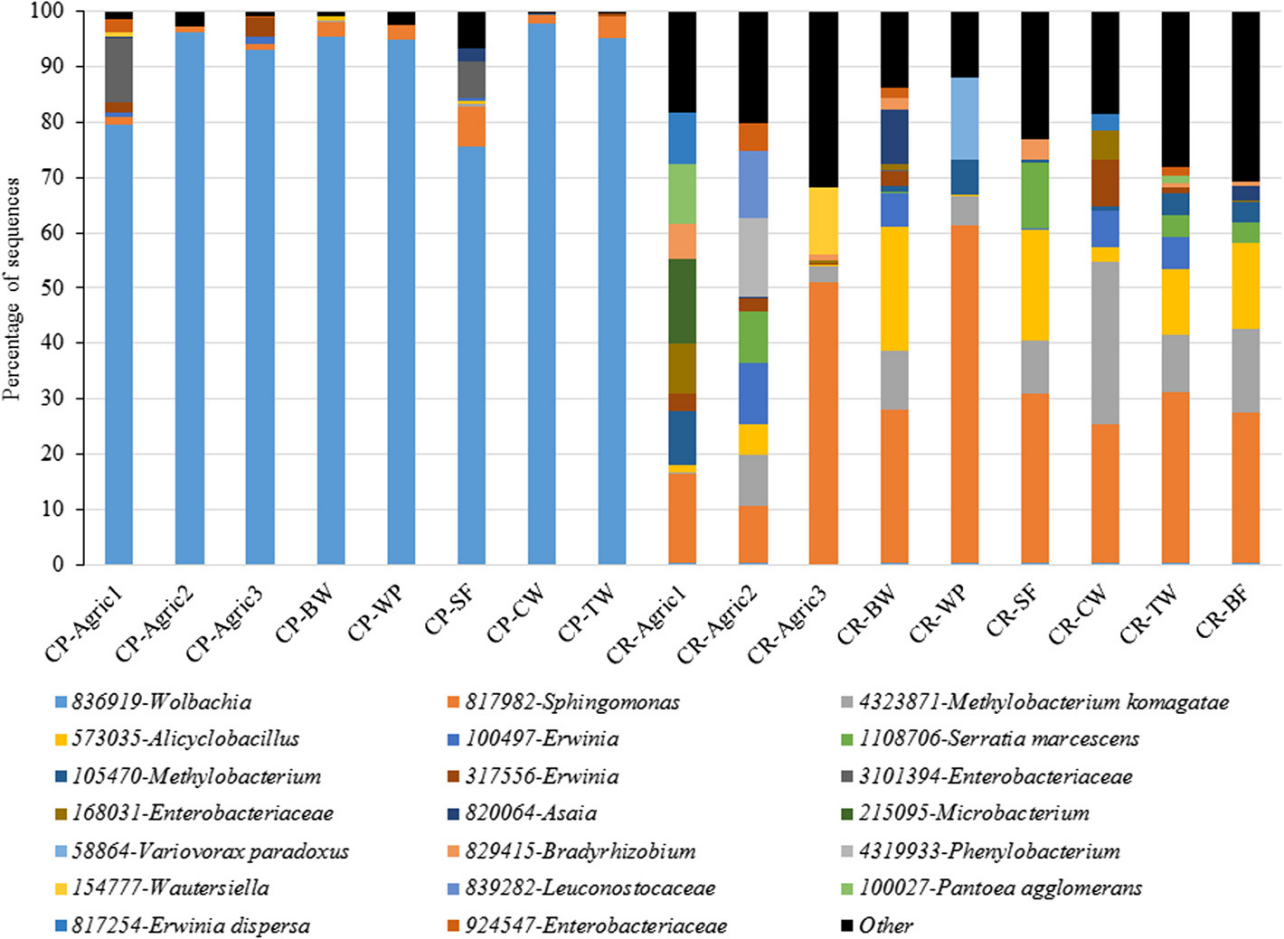
Relative abundance of the top 20 bacterial OTUs in *Cx. pipiens* and *Cx. restuans* samples from different study sites. (CP = *Cx. pipiens*, CR = *Cx. restuans*, Agric = Agriculture, BW = Busey Wood, WP = Weaver Park, SF = South Farms, CW = Collins Woods, TW = Trelease Woods, and BF = Brownfield Woods)

To determine the expected richness in each of the two mosquito species, we calculated Chao1 estimator based on OTUs abundance (Table 2). Overall, we were able to detect 80 % (range 62–100 %) and 69 % (54–79 %) of expected number of OTUs in *Cx. pipiens* and *Cx. restuans*, respectively indicating an underestimation of gut microbial diversity. On average, we estimated that *Cx. pipiens* contains 11 bacterial OTUs (range 5–15) while *Cx. restuans* contains 22 bacterial OTUs (range 18–29). In addition to the total number of OTUs and the Chao1 richness estimator to compare sample microbial diversity, we calculated the Shannon index to measure OTU richness and homogeneity in mosquito samples. These estimates indicated that bacterial OTUs were significantly more diverse and equitably distributed in *Cx. restuans* compared to *Cx. pipiens* in all study sites (Table 2). The top 20 bacterial OTUs were heterogeneously distributed across study sites accounting for 93–99.5 % of total microbiota in *Cx. pipiens* and 69–87 % of total microbiota in *Cx. restuans* (Fig. 3). Lower OTU diversity and evenness in *Cx. pipiens* was driven by the dominance of *Wolbachia* spp. (OTU836919), which accounted for between 76 % and 98 % of total bacteria in this mosquito species (Fig. 3). Overall, each of the remaining OTUs accounted for less than 0.75 % of total bacteria in *Cx. pipiens* with exception of OTU817982 (*Sphingomonas* spp.) and OTU3101394 (*Enterobacteriaceae*), which accounted for 2.8 % (range 1.1–7.3 %) and 2.3 % (0–12 %) of total bacteria respectively. In contrast, the most dominant OTU in *Cx. restuans* was OTU817982-*Sphingomonas* spp. (31 %) and was more abundant in two study sites, Weaver (61 %) and agriculture site 3 (51 %), and less common in agriculture site 2 (10 %, Fig. 3). The second and third most abundant OTUs in *Cx. restuans* were OTU4323871 (*Methylobacterium* spp.) and OTU573035 (*Alicyclobacillus* spp.), which accounted for 10 % (range 0.4–29 %) and 9 % (range 0.1–23 %) of total bacteria, respectively (Fig. 3). Overall, only 12 OTUs had an overall relative abundance equal to, or greater than, 1 % (Fig. 3).

**Table 2:** Biodiversity (Shannon, evenness) and richness estimators of *Cx. pipiens* and *Cx. restuans* microbiota (± standard error)

Mosquito species	Study site	Shannon	Equitability	Observed species	Chao1
*Cx. pipiens*	Agriculture1	0.65 ± 0.14	0.23 ± 0.05	7.36 ± 0.62	9.45 ± 0.88
	Agriculture2	0.24 ± 0.11	0.09 ± 0.04	6.33 ± 1.20	8.00 ± 2.65
	Agriculture3	0.36 ± 0.26	0.11 ± 0.07	6.20 ± 1.98	8.20 ± 2.82
	Busey Woods	0.32 ± 0.26	0.11 ± 0.08	6.50 ± 2.50	8.00 ± 4.00
	Weaver Park	0.29 ± 0.09	0.10 ± 0.03	7.20 ± 0.85	11.55 ± 2.23
	South Farms	0.75 ± 0.16	0.21 ± 0.04	10.41 ± 1.14	14.78 ± 2.33
	Collins Woods	0.19 ± 0.00	0.06 ± 0.00	9.00 ± 0.00	9.60 ± 0.00
	Trelease Woods	0.33 ± 0.00	0.14 ± 0.00	5.00 ± 0.00	5.00 ± 0.00
	Total	0.53 ± 0.07	0.17 ± 0.02	8.14 ± 0.54	11.33 ± 1.04
*Cx. restuans*	Agriculture1	2.23 ± 0.17	0.55 ± 0.01	17.33 ± 4.10	23.58 ± 8.25
	Agriculture2	1.32 ± 0.26	0.36 ± 0.07	12.57 ± 1.57	18.05 ± 2.20
	Agriculture3	1.66 ± 0.42	0.39 ± 0.10	18.80 ± 1.07	28.37 ± 3.17
	Busey Woods	1.46 ± 0.16	0.38 ± 0.04	14.60 ± 1.17	18.37 ± 1.61
	Weaver Park	1.16 ± 0.09	0.29 ± 0.02	17.20 ± 1.46	25.27 ± 2.82
	South Farms	1.66 ± 0.15	0.42 ± 0.03	16.17 ± 1.54	20.45 ± 2.33
	Collins Woods	1.20 ± 0.20	0.31 ± 0.06	15.67 ± 1.52	28.92 ± 5.01
	Trelease Woods	1.72 ± 0.19	0.46 ± 0.05	14.64 ± 1.17	20.26 ± 2.96
	Brownfield	1.63 ± 0.18	0.41 ± 0.03	15.82 ± 1.68	25.64 ± 4.31
	Total	1.56 ± 0.07	0.40 ± 0.02	15.49 ± 0.55	22.37 ± 1.24

### The two mosquito species have different bacterial community structure

**Fig. 4:**
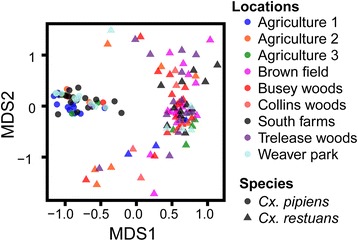
Nonmetric Multidimensional Scaling (NMDS) ordination displaying microbiome communities of *Cx. pipiens* and *Cx. restuans*. Microbiomes were distinct between the two mosquito species in each study site

To determine if bacterial communities differed between the two mosquito species and across study sites, nonparametric multivariate community analyses were conducted. MRPP results indicated that midgut bacterial communities of the two mosquito species across all study sites were significantly different with those of *Cx. pipiens* being more homogeneous compared to those of *Cx. restuans* (Table 3, Fig. 4). Indicator Species Analysis (ISA) was used to characterize the bacterial OTUs that were strongly associated with one mosquito species over the other. One and 8 indicator species were identified for *Cx. pipiens* and *Cx. restuans* respectively (Table 4). *Wolbachia* (OTU836919), the only indicator species identified for *Cx. pipiens* had an indicator value close to 1. Only three of 8 indicator species identified for *Cx. restuans* had an indicator value greater than 0.6 (Table 4). These included OTU817982 (*Sphingomonas* spp.), OTU573035 (*Alicyclobacillus* spp.), and OTU4323871 (*Methylobacterium komagatae*, Table 4).

**Table 3:** MRPP results showing differences in bacterial communities between *Cx. pipiens* and *Cx. restuans* from each study site. Only 5 of 9 study sites are presented because the remaining sites had either few or no *Cx. pipiens* samples to facilitate meaningful comparisons

*Location*	N(*Cp* vs *Cr*)	Ad (*Cp* vs. *Cr*)	T	A^3^	*P* ^4^
Agriculture 1	11 vs. 3	0.31 vs. 0.95	-5.47	0.22	0.001
Agriculture 2	3 vs. 7	0.61 vs. 0.97	-5.78	0.24	0.001
Agriculture 3	5 vs. 5	0.13 vs. 0.69	-5.62	0.44	0.002
Busey woods	2 vs. 15	0.09 vs. 0.78	-5.25	0.44	0.003
South farms	17 vs. 12	0.38 vs. 0.74	-13.55	0.26	0.000002
Weaver Park	10 vs. 5	0.07 vs. 0.53	-8.90	0.57	0.00007

*N* number of *Cx. pipiens* (*Cp*) and *Cx. restuans* (*Cr*) samples

*Ad* average within group distances for the mosquito species

*T* test statistic describing separation between groups

*A* chance-corrected within group agreement as log_10_

**Table 4:** Bacterial OTUs that characterize *Cx. pipiens* and *Cx. restuans* mosquitoes

	OTU ID	Phylum^a^	Class^b^	Species/Other^c^	Indicator value^d^	*P*
*Cx. pipiens*	836919	P	A	*Wolbachia* spp	99.6	0.0002
*Cx. restuans*	817982	P	A	*Sphingomonas* spp.	82.5	0.0002
	573035	F	B	*Alicyclobacillus* spp.	60.6	0.0002
	4323871	P	A	*Methylobacterium komagatae*	66.8	0.0002
	4363508	P	A	Sphingomonadaceae	54.0	0.0002
	17329	P	A	Sphingomonadaceae	54.2	0.0002
	4344371	P	A	*Sphingomonas* spp.	46.2	0.0002
	4378646	P	A	Sphingomonadaceae	45.0	0.0002
	4396025	P	A	*Sphingomonas* spp.	40.4	0.0002

^a^Phyla abbreviations: *P* Proteobacteria, *F* Firmicutes

^b^Class abbreviations: *A* Alphaproteobacteria, *B* Bacilli, *G* Gammaproteobacteria

^c^ The lowest classification based on greengenes database

^d^Indicator value computed by Indicator Species Analysis

MRPP analyses revealed that bacterial communities did not differ significantly between *Cx. pipiens* from different study sites (data not shown). In addition, only 4 of 36 (11 %) study site combinations had significantly different bacterial communities for *Cx. restuans* indicating weak effects of study site on bacterial community structure (Table 5).

**Table 5:** MRPP results showing differences in bacterial communities of *Cx. restuans* mosquitoes from different study sites

Location	Ad	N	T	A	*P*
Agriculture 2 vs. Agriculture 3	0.97 vs. 0.69	7 vs. 5	-2.27	0.05	0.03
Agriculture 2 vs. Weaver Park	0.97 vs. 0.54	7 vs. 5	-2.93	0.10	0.02
Weaver Park vs. South farms	0.54 vs. 0.79	5 vs. 12	-2.45	0.06	0.03
Weaver vs. Brownfield	0.54 vs. 0.77	5 vs. 17	-2.80	0.04	0.02

*Ad* average within group distances for the mosquito species

*N* sample size

*T* test statistic describing separation between groups

*A* chance-corrected within group agreement as log_10_

Results are only shown for groups that were significantly different at *P* ≤ 0.05

## Discussion

This study provides the first comprehensive analysis of the microbial consortia of field-collected populations of *Cx. pipiens* and *Cx. restuans*, the primary vectors of WNV in northeastern and Midwestern United States [41]. Although both mosquito species were dominated by members of phylum *Proteobacteria* as reported for other mosquito species [2, 4, 6, 25], their bacterial composition and community structure differed significantly with *Cx. restuans* hosting more diverse and evenly distributed microbial communities compared to *Cx. pipiens*. In particular, we found *Wolbachia* (OTU836919) and *Sphingomonas* (OTU817982) to be the most abundant and the best indicator species for *Cx. pipiens* and *Cx. restuans*, respectively.

*Wolbachia* (OTU836919) was present in 98 % of all mosquito samples and accounted for 76–98 % of total microbiota in *Cx. pipiens* and 0.2–0.4 % total microbiota in *Cx. restuans. Wolbachia* are maternally inherited bacterial endosymbionts that infect numerous invertebrates [42–45] and are known to cause reproductive alterations in their hosts including feminization, parthenogenesis, male killing, and cytoplasmic incompatibility [46]. In *Cx. pipiens*, *Wolbachia* causes partial or complete cytoplasmic incompatibility (CI) between infected males and uninfected females or females infected by incompatible strains of *Wolbachia* [47, 48]. This manipulation confers relative fitness advantage to infected females allowing *Wolbachia* to rapidly invade host populations [49]. These properties combined with recent reports that certain strains of *Wolbachia* can shorten the mosquito lifespan [50], reduce their blood feeding success [51], and inhibit their ability to transmit a range of pathogens including dengue, Chikungunya, and malaria [52–56] has generated interest in utilizing *Wolbachia* for the control of mosquito-borne diseases. However, the utility of this endosymbiont for the control of *Culex*-borne viruses such as WNV requires further investigations because certain *Wolbachia* strains may enhance or inhibit replication of WNV in some mosquito species [57, 58]. Therefore, it is likely that *Wolbachia* infection contributes to the spatial and temporal heterogeneity in WNV transmission that is common in endemic areas.

*Wolbachia* occurs in most populations of *Cx. pipiens* [44, 59, 60] and were recently detected in three *Cx. restuans* individuals [61]. Thus our results suggest that *Wolbachia* is more widespread in *Cx. restuans* than previously reported, and is a widespread and dominant bacterial species in *Cx. pipiens*. It is unclear why the relative abundance of this endosymbiont was low in *Cx. restuans*. Potential explanations include differences in the physiology of the two mosquito species where environmental conditions in *Cx. restuans* limit proliferation of *Wolbachia* or competition with the diverse bacterial communities that were identified in this mosquito species. The latter has been demonstrated in *Anopheles gambiae* Giles and *An. stephensi* Liston where native microbiota impeded replication and maternal transmission of *Wolbachia* [62].

Unlike *Cx. pipiens*, the most abundant bacterial species in *Cx. restuans* was *Sphingomonas* spp. (OTU817982), which accounted for 31 % of total microbiota. This OTU occurred in 90 % of all mosquito samples albeit at low abundance in *Cx. pipiens*. These findings suggest that this bacterium has either established a symbiotic association with the two mosquito species or is widespread in nature. Existing literature indicates that members of the genus *Sphingomonas* are widely distributed in both terrestrial and aquatic environments primarily due to their unique abilities to survive under low concentrations of nutrients and to metabolize a wide variety of carbon sources [63]. *Sphingomonas* spp. are also common in insects and have been isolated in mosquito species from the genera *Anopheles*, *Culex*, *Aedes* and *Mansonia* [2, 64, 65]. Future studies should investigate their role in mosquito biology.

The majority of bacterial OTUs were sparsely distributed among individuals of the two mosquito species likely due to genetic factors and/or inter-individual variations in diet [1, 2, 66]. This variation may partly account for population and species level variation in vector competence that is commonly observed in nature because certain bacterial species are known to increase [58, 67] or reduce [7, 16–18] vector susceptibility to pathogens. Further studies are needed to investigate the role of identified microbiota on the biology of the two mosquito species including susceptibility to pathogens such as WNV.

The two mosquito species had distinct bacterial profiles although they were collected from the same study sites, are known to utilize the same aquatic habitats for larval development, and tend to blood feed primarily on avian hosts [68–70]. The mechanisms underlying these variations are unclear but we can offer some hypotheses. First, differences in larval foraging and adult sugar feeding may lead to differential exposure of the two mosquito species to microbial communities. Although the larval feeding behavior of *Cx. pipiens* and *Cx. restuans* is not well understood, variation in larval feeding behavior of *Culex* mosquitoes has been reported before [71]. Moreover, all mosquitoes feed on plant sugars and each mosquito species may show preference for specific plants [72, 73]. Previous studies have documented a marked difference in bacterial composition between plant species [74, 75]. Therefore, if the two mosquito species differ in their preferred sugar-meal sources, they may be exposed to different bacterial communities. Second, the two mosquito species may possess different physiologies and thereby inherent differences in the ability to support proliferation and survival of different bacterial species. Third, *Cx. pipiens* and *Cx. restuans* show temporal variation in their distribution with *Cx. restuans* populations peaking earlier (June-July) than those of *Cx. pipiens* [76]. Temporal variations in the collection of the two mosquito species may partly account for differences in microbial communities given that the relative abundance of bacterial species may vary seasonally [77].

Lastly, although gravid traps are specifically designed to collect gravid females seeking suitable oviposition sites, they also collect a small proportion of host-seeking females [78]. These may consist of newly emerged females as well as older females that may have previously acquired a sugar meal and/or a blood meal. Previous research suggests that newly emerged adults have a more diverse microbial community relative to sugar fed or blood fed mosquitoes [1]. Although we used mosquito samples that were not visibly blood-engorged, we did not record their physiological status. We also did not have prior knowledge of whether the small fraction of host-seeking females that may have been collected in our study had acquired a blood meal or a sugar meal before they were captured. Nevertheless, there is no documented evidence that gravid traps are more effective at collecting gravid females of *Cx. pipiens* compared to those of *Cx. restuans* and vice versa. Thus we cannot attribute the differences in microbiota between the two mosquito species to trap bias in the collection of gravid versus host-seeking individuals of the two species.

## Conclusions

Our study is the first to provide a comprehensive description of microbial communities associated with two of the most important vectors of WNV in the United States. We found that the two mosquito species harbor distinct microbial communities that are heterogeneously distributed between individuals. *Wolbachia* was the dominant bacterial species in *Cx. pipiens* while more diverse and variable microbial communities were observed in *Cx. restuans*. The results expand the range of mosquito species whose microbial communities have been characterized and will allow further studies to characterize the function of different bacterial species on the biology of the two vector species and their contribution to vectorial capacity. This knowledge will inform identification of microbial communities that can be used to block transmission of *Culex*-borne pathogens such as WNV.

### Data availability

All relevant data are either within the paper and its Additional files or held in a public repository at NCBI under project number SUB1053826. The accession numbers for 16S amplicons from whole *Culex pipiens* and *Culex restuans* mosquitoes are SAMN03968624-SAMN03968758.

## Additional files

Additional file 1: Table S1. GPS coordinates for the nine study sites and the number of *Culex* spp. mosquitoes collected from each site. (DOCX 12 kb) Additional file 2: Appendix S1. Description of Fluidign Access Array Amplification. (DOCX 14 kb) Additional file 3: Figure S1. Relative abundance of the top 20 bacterial families in *Cx. pipiens* and *Cx. restuans* samples from different study sites. (CP = *Cx. pipiens*, CR = *Cx. restuans*, Agric = Agriculture, BW = Busey Wood, WP = Weaver Park, SF = South Farms, CW = Collins Woods, TW = Trelease Woods, and BF = Brownfield Woods). Results were generated using reverse primer reads. (TIF 72 kb) Additional file 4: Figure S2. Relative abundance of the top 20 bacterial OTUs in *Cx. pipiens* and *Cx. restuans* samples from different study sites. (CP = *Cx. pipiens*, CR = *Cx. restuans*, Agric = Agriculture, BW = Busey Wood, WP = Weaver Park, SF = South Farms, CW = Collins Woods, TW = Trelease Woods, and BF = Brownfield Woods). Results were generated using reverse primer reads. (TIF 73 kb) Additional file 5: Table S2. Biodiversity (Shannon, evenness) and richness estimators of *Cx. pipiens* and *Cx. restuans* microbiota (± standard error). The data was generated using reverse reads. (DOC 50 kb) Additional file 6: Table S3. Taxonomy of bacterial OTUs that were shared between *Cx. pipiens* and *Cx. restuans* or were unique to either species. (XLSX 17 kb)
