# Supplementary material for: Culex pipiens and Culex restuans mosquitoes harbor distinct microbiota dominated by few bacterial taxa
Source: Parasit Vectors. 2016 Jan 13;9:18. doi: 10.1186/s13071-016-1299-6 (PMC4712599; doi:10.1186/s13071-016-1299-6)
Supplement: Additional file 1: Table S1. — GPS coordinates for the nine study sites and the number of Culex spp. mosquitoes collected from each site. (DOCX 12 kb) [file 13071_2016_1299_MOESM1_ESM.docx]

S1_Table: GPS coordinates for the nine study sites and the number of *Culex* spp. mosquitoes collected.

| **Location** | **Latitude** | **Longitude** | **No. Mosquitoes** |
| --- | --- | --- | --- |
| Agriculture 1 | 40^o^05'34''N | 88^o^14'32''W | 44 |
| Agriculture 2 | 40^o^10'17''N | 88^o^08'34''W | 163 |
| Agriculture3 | 39^o^59'45''N | 88^o^16'04''W | 62 |
| Busey Woods | 40^o^07'12''N | 88^o^12'27''W | 58 |
| Weaver Park | 40^o^06'42''N | 88^o^10'37''W | 46 |
| South Farms | 40^o^05'05''N | 88^o^12'50''W | 114 |
| Collins Woods | 40 ^o^08'18''N | 88^o^02' 00''W | 28 |
| Trelease Woods | 40^o^07'46''N | 88^o^08'34''W | 134 |
| Brownfield | 40^o^07'42''N | 88^o^10'27''W | 40 |
