# Supplementary material for: Culex pipiens and Culex restuans mosquitoes harbor distinct microbiota dominated by few bacterial taxa
Source: Parasit Vectors. 2016 Jan 13;9:18. doi: 10.1186/s13071-016-1299-6 (PMC4712599; doi:10.1186/s13071-016-1299-6)
Supplement: Additional file 2: Appendix S1. — Description of Fluidign Access Array Amplification. (DOCX 14 kb) [file 13071_2016_1299_MOESM2_ESM.docx]

**Fluidigm Access Array Amplification**

Prior to amplification, all DNA samples were measured on a Qubit (Life Technologies) using the High Sensitivity DNA Kit. Samples were diluted to 2 ng/µl concentrations. A mastermix for amplification was prepared using the Roche High Fidelity Fast Start Kit and 20x Access Array loading reagent according to Fluidigm protocols. For each sample the following reagents were combined:

0.5 µl -10X FastStart Reaction Buffer without MgCl_2_

0.9 µl -25 mM MgCl_2_

0.25 µl – DMSO

0.1 µl -10 mM PCR grade Nucleotide Mix

0.05 µl -5 U/µl FastStart High Fidelity Enzyme Blend

0.25 µl – 20X Access Array Loading Reagent

0.95 µl – Water

Mastermix was aliquoted to 48 wells of a PCR plate. To each well, 1 µl DNA sample and 1 µl Fluidigm Illumina linkers with unique barcode were added. In a separate plate, primer pairs were prepared and aliquoted. 20X primer solutions were prepared by adding 2 µl of each forward and reverse primer, 5 µl of 20X Access Array Loading Reagent and water to a final volume of 100 µl.

Primer sequences for 16S V3-V5 hypervariable region were synthesized by IDT Corp. (Coralville, IA) and are listed below:

**Fluidigm CS1 Adapter**

**Fluidigm CS2 Adapter**

16S V3-V5-F357 **ACACTGACGACATGGTTCTACA**CCTACGGGAGGCAGCAG

16S V3-V5 R926 **TACGGTAGCAGAGACTTGGTCT**CCGTCAATTCMTTTRAGT

Four µl of sample was loaded in the sample inlets and 4 µl of primer loaded in primer inlets of a previously primed Fluidigm 48.48 Access Array IFC. The IFC was placed in an AX controller (Fluidigm Corp.) for microfluidic loading of all primer/sample combinations. Following the loading stage, the IFC plate was loaded on the Fluidigm Biomark HD PCR machine and samples were amplified using the following Access Array cycling program without imaging:

**PCR Stages Number of Cycles**

50ºC 2 minutes 1

70ºC 20 minutes 1

95ºC 10 minutes 1

95ºC 15 seconds

60ºC 30 seconds

72ºC 1 minute 10

95ºC 15 seconds

80ºC 30 seconds

60ºC 30 seconds

72ºC 1 minute 2

95ºC 15 seconds

60ºC 30 seconds

72º 1 minute 8

95ºC 15 seconds

80ºC 30 seconds

60ºC 30 seconds

72ºC 1 minute 2

95ºC 15 seconds

60ºC 30 seconds

72ºC 1 minute 8

95ºC 15 seconds

80ºC 30 seconds

60ºC 30 seconds

72ºC 1 minute 5

Following amplification, 2 µl of Fluidigm Harvest Buffer was loaded in the sample inlets and loaded on the AX controller for harvesting PCR products. Harvested product was then transferred to a new 96 well plate quantified on a Qubit fluorimeter (Thermo-Fisher) and stored at -20°C. All samples were run on a Fragment Analyzer (Advanced Analytics, Ames, IA) and amplicon regions and expected sizes confirmed. Samples were then pooled in equal amounts according to product concentration. The pooled products were then size selected on a 2% agarose E-gel (Life Technologies) and extracted from the isolated gel slice with QIAquick gel extraction kit (QIAGEN). Cleaned size selected products were run on an Agilent Bioanalyzer to confirm appropriate profile and determination of average size.
