# Supplementary figures and images for: Culex pipiens and Culex restuans mosquitoes harbor distinct microbiota dominated by few bacterial taxa
Source: Parasit Vectors. 2016 Jan 13;9:18. doi: 10.1186/s13071-016-1299-6 (PMC4712599; doi:10.1186/s13071-016-1299-6)

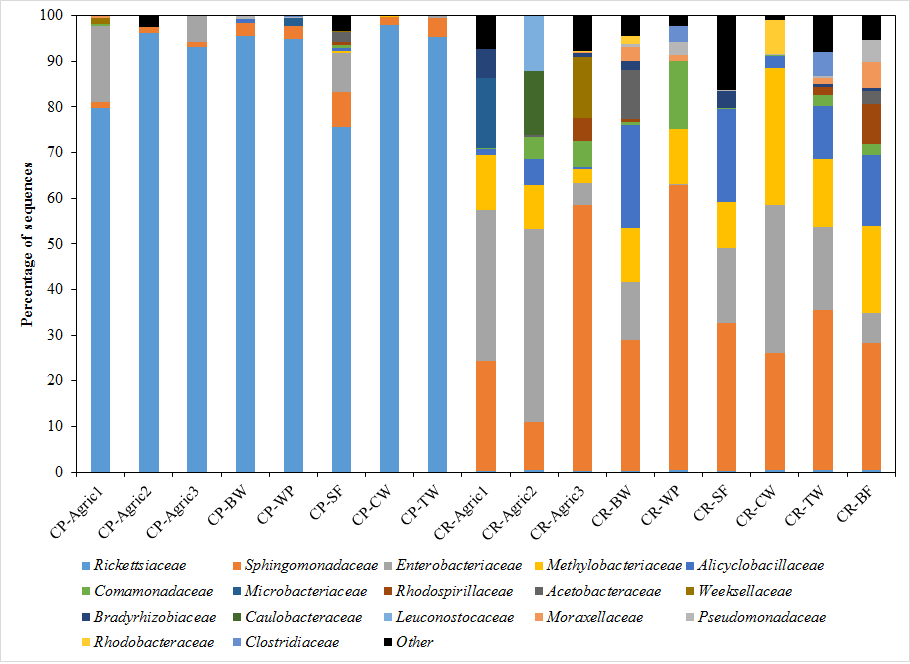

Supplement: Additional file 3: Figure S1. — Relative abundance of the top 20 bacterial families in Cx. pipiens and Cx. restuans samples from different study sites. (CP = Cx. pipiens, CR = Cx. restuans, Agric = Agriculture, BW = Busey Wood, WP = Weaver Park, SF = South Farms, CW = Collins Woods, TW = Trelease Woods, and BF = Brownfield Woods). Results were generated using reverse primer reads. (TIF 72 kb) [file 13071_2016_1299_MOESM3_ESM.tif]

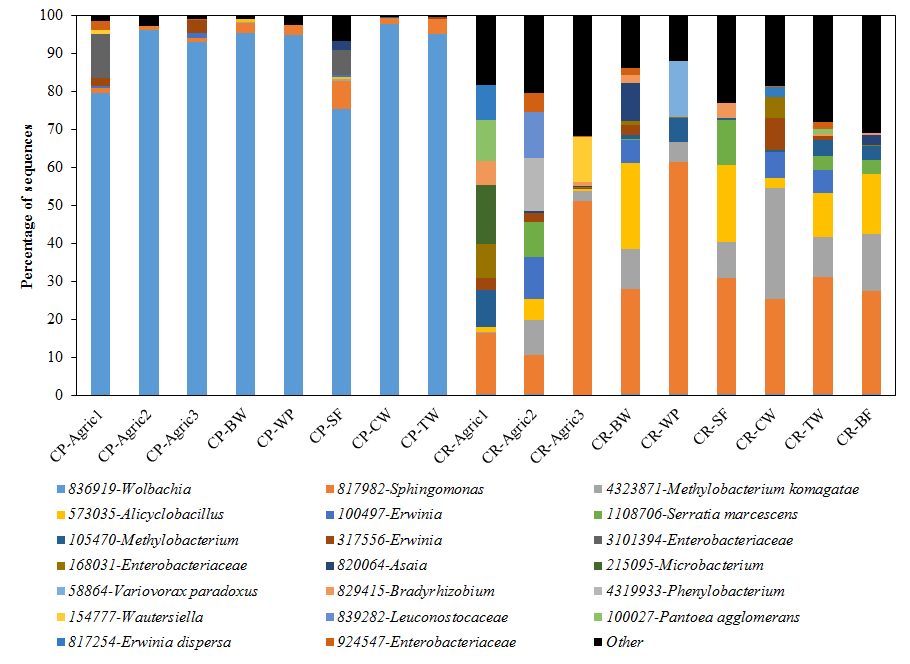

Supplement: Additional file 4: Figure S2. — Relative abundance of the top 20 bacterial OTUs in Cx. pipiens and Cx. restuans samples from different study sites. (CP = Cx. pipiens, CR = Cx. restuans, Agric = Agriculture, BW = Busey Wood, WP = Weaver Park, SF = South Farms, CW = Collins Woods, TW = Trelease Woods, and BF = Brownfield Woods). Results were generated using reverse primer reads. (TIF 73 kb) [file 13071_2016_1299_MOESM4_ESM.tif]
