# Supplementary material for: Culex pipiens and Culex restuans mosquitoes harbor distinct microbiota dominated by few bacterial taxa
Source: Parasit Vectors. 2016 Jan 13;9:18. doi: 10.1186/s13071-016-1299-6 (PMC4712599; doi:10.1186/s13071-016-1299-6)
Supplement: Additional file 5: Table S2. — Biodiversity (Shannon, evenness) and richness estimators of Cx. pipiens and Cx. restuans microbiota (± standard error). The data was generated using reverse reads. (DOC 50 kb) [file 13071_2016_1299_MOESM5_ESM.doc]

S2-Table: Biodiversity (Shannon, evenness) and richness estimators of *Cx. pipiens* and *Cx. restuans* microbiota (± standard error). The data was generated using reverse reads.

| Species | Study site | Shannon | Equitability | Observed  species | Chao1 |
| --- | --- | --- | --- | --- | --- |
| *Cx pipiens* | Agriculture1 | 0.60 ± 0.14 | 0.19 ± 0.05 | 14.36 ± 4.08 | 19.76 ± 6.18 |
|  | Agriculture2 | 0.12 ± 0.04 | 0.05 ± 0.01 | 5.67 ± 0.67 | 6.17 ± 0.73 |
|  | Agriculture3 | 0.39 ± 0.31 | 0.08 ± 0.05 | 12.80 ± 8.47 | 13.65 ± 9.25 |
|  | Busey Woods | 0.22 ± 0.21 | 0.06 ± 0.06 | 7.00 ± 5.00 | 7.00 ± 5.00 |
|  | Weaver Woods | 0.22 ± 0.07 | 0.07 ± 0.02 | 7.50 ± 1.28 | 9.83 ± 1.88 |
|  | South Farms | 0.72 ± 0.19 | 0.18 ± 0.04 | 12.82 ± 2.34 | 15.45 ± 3.18 |
|  | Collins Woods | 0.10 ± 0.00 | 0.03 ± 0.00 | 9.00 ± 0.00 | 14.00 ± 0.00 |
|  | Trelease Woods | 0.24 ± 0.00 | 0.07 ± 0.00 | 12.00 ± 0.00 | 15.33 ± 0.00 |
|  | Total | 0.48 ± 0.08 | 0.13 ± 0.02 | 11.34 ± 1.48 | 14.17 ± 2.01 |
| *Cx. restuans* | Agriculture1 | 2.68 ± 0.76 | 0.53 ± 0.14 | 32.00 ± 3.00 | 38.38 ± 2.63 |
|  | Agriculture2 | 2.27 ± 0.59 | 0.46 ± 0.09 | 29.67 ± 6.92 | 33.81 ± 7.24 |
|  | Agriculture3 | 2.11 ± 0.26 | 0.46 ± 0.06 | 26.00 ± 3.59 | 31.36 ± 7.68 |
|  | Busey Woods | 2.19 ± 0.32 | 0.47 ± 0.05 | 24.69 ± 4.22 | 26.53 ± 4.61 |
|  | Weaver Woods | 1.79 ± 0.21 | 0.46 ± 0.03 | 17.50 ± 4.44 | 17.62 ± 4.51 |
|  | South Farms | 2.20 ± 0.14 | 0.51 ± 0.03 | 21.25 ± 2.17 | 27.84 ± 5.58 |
|  | Collins Woods | 2.81 ± 0.75 | 0.57 ± 0.10 | 31.00 ± 10.00 | 33.03 ± 11.78 |
|  | Trelease Woods | 2.48 ± 0.29 | 0.57 ± 0.05 | 23.12 ± 3.67 | 26.38 ± 4.83 |
|  | Brownfield | 2.21 ± 0.32 | 0.51 ± 0.05 | 20.45 ± 3.24 | 21.39 ± 3.34 |
|  | Total | 2.26 ± 0.12 | 0.50 ± 0.02 | 23.93 ± 1.54 | 27.00 ± 1.91 |
